# Bulk Data Dissemination in Low Power Sensor Networks: Present and Future Directions

**DOI:** 10.3390/s17010156

**Published:** 2017-01-14

**Authors:** Zhirong Xu, Tianlei Hu, Qianshu Song

**Affiliations:** 1 Collge of Computer Science, Zhejiang University, Zhejiang 310027, China; 2 Zhejiang Association for Science and Technology, Zhejiang 310003, China; negosong@163.com

**Keywords:** bulk dissemination protocols, survey, low power, sensor networks

## Abstract

Wireless sensor network-based (WSN-based) applications need an efficient and reliable data dissemination service to facilitate maintenance, management and data distribution tasks. As WSNs nowadays are becoming pervasive and data intensive, bulk data dissemination protocols have been extensively studied recently. This paper provides a comprehensive survey of the state-of-the-art bulk data dissemination protocols. The large number of papers available in the literature propose various techniques to optimize the dissemination protocols. Different from the existing survey works which separately explores the building blocks of dissemination, our work categorizes the literature according to the optimization purposes: Reliability, Scalability and Transmission/Energy efficiency. By summarizing and reviewing the key insights and techniques, we further discuss on the future directions for each category. Our survey helps unveil three key findings for future direction: (1) The recent advances in wireless communications (e.g., study on cross-technology interference, error estimating codes, constructive interference, capture effect) can be potentially exploited to support further optimization on the reliability and energy efficiency of dissemination protocols; (2) Dissemination in multi-channel, multi-task and opportunistic networks requires more efforts to fully exploit the spatial-temporal network resources to enhance the data propagation; (3) Since many designs incur changes on MAC layer protocols, the co-existence of dissemination with other network protocols is another problem left to be addressed.

## 1. Introduction

The recent advances of the low-power wireless communication and computation has led to the development of low power wireless sensor networks (WSNs) for the Internet of Things (IoT). A typical WSN consists of a (large) number of resource constrained sensor nodes equipped with low power sensors, CPUs and radios. WSN-based applications covers large-area monitoring [1,2,3], smart grid [4], health care [5], smart homes [6], green buildings [7], etc. In most of these applications, WSNs are deployed for a long term operation without manual intervention. Therefore, maintenance and management are critical for WSNs. To this end, data dissemination is the key enabling protocol for delivering commands and software updates to the entire network.

There are several basic requirements for dissemination protocols in WSNs.

- Reliability. Since it is often used for distribute commands and code updates to all network nodes, 100% reliability is typically required (i.e., all nodes should receive all the transmitted data).
- Scalability. For maintaining a WSN, there are typically multiple parameters to maintain, e.g., sensing data type, neighbor table, etc. Hence the framework should be scalable to support multi-object and large scale dissemination.
- Efficiency. Due to the resource-constrained nature of WSN nodes, dissemination should be done in a transmission/energy efficient way.

Most of the recent works on bulk data dissemination have been devoted to meet the above requirements [8,9,10,11,12]. We categorize the literature according to the optimization goals: (1) Works for optimizing reliability; (2) works for optimizing scalability. (3) works for optimizing (transmission/energy) efficiency. In these works, some novel observations and techniques are exploited, such as link correlation [13], constructive interference [14], network coding [15], etc.

It is worth noting that the recent advances in wireless communications shed light on further optimizations of data dissemination in low power wireless sensor networks. Specifically,

1. Cross-technology interference/communications. As many IoT applications of sensor networks are deployed in an indoor environment (e.g., smart home [6], body sensor network [16], etc.), other wireless devices such as WiFi and BlueTooth may cause fierce cross-technology interference (CTI) to WSNs. The recent advances in reducing CTI and enabling cross-technology communication can potentially support further optimization on dissemination in low power WSNs.
2. Coding techniques. Network coding is an important means to improve the transmission efficiency in dissemination. However, the complexity and the corresponding coding delay have always been an obstacle for the utilization in dissemination. Some novel coding designs such as [17] achieves considerable improvement in the coding delay, which is a promising alternative to improve the dissemination performance.
3. Constructive Interference. Constructive Interference [14,18] is a novel communication technique that supports highly efficient data transmissions in multi-sender multi-receiver scenario, which can be potentially used for optimizing dissemination.

In this paper, we provide a comprehensive review of the recent developments in the field of dissemination. For each category of different optimization goals, we summarize and compare the key ideas and techniques. Besides, the design tradeoffs and limitations of the existing works are discussed in detail. We also explore the future directions with a special consideration of the recent advances in (1) wireless cross-technology communications (2) network coding and (3) constructive interference.

The remainder of this paper is organized as follows: Section 2 describes the recent advances on bulk data dissemination. We further categorize them into four sub-categories according to the optimization goals. Section 3 present the potential directions for data dissemination, with a special focus on exploiting the recent advances in wireless communications. Section 4 concludes this paper.

## 2. Bulk Data Dissemination

In this section, we first discuss the application scenarios of bulk data dissemination and then summarize the corresponding requirements. With the summarized requirements, we present the taxonomy and discussions.

### 2.1. Application Scenarios

Bulk data dissemination is often used for reprogramming, network updates, surveillance video distribution, etc. For example, in deploying the large-scale sensor network system—GreenOrbs [19] during December 2010 through April 2011, the requirement of software upgrade is regularly faced. According to [20], the software version increased from version 158 to version 285, where each version requires at least one run of bulk data dissemination. The data (or code) object varies from 15 KB to 40 KB. Considering that the typical memory is only 48 KB, the data objects are relatively large.

### 2.2. Key Requirements for Bulk Data Dissemination

Considering the application scenarios, the characteristics of WSNs and the dissemination protocol, we summarize the requirements for bulk data dissemination as follows.

#### 2.2.1. Reliability

To support network reprogramming, surveillance data distribution, etc., reliable dissemination to all nodes is required [21]. The reliability here is strict in that all of the data object should be received in its entirety. High loss rates common to wireless sensor networks force the use of different solutions than those used for traditional wired network models [22].

#### 2.2.2. Scalability

Scalability is important because many of the sensor network applications run on tens, hundreds or even thousands of sensor nodes [19,23,24]. The scalability concerns are exacerbated by the bandwidth constraints, compared say to the Internet-scale distributed systems. For example, the wireless PHY/MAC protocol for sensor nodes IEEE 802.15.4, provides a maximum bandwidth of 250 kbps. Therefore, the primary concern on the scalability of sensor networks is about reducing the amount of communication to efficiently use the scarce bandwidth [25]. As a result, scalability is also a crucial issue for bulk data dissemination in sensor networks. The problem of scalability concerns mainly two aspects: (i) Network size. The dissemination protocol should work well for large-scale sensor networks which may consists of hundreds or thousands of sensor nodes; (ii) Network density. The dissemination protocol should work well with high network density.

#### 2.2.3. Transmission & Time Efficiency

Considering the network might not work normally during bulk data dissemination, the delay and transmission of dissemination needs to be minimized. There are two main challenges for transmission and time efficiency in bulk data dissemination: (i) Resource constraints. Sensor nodes are commonly small in size with limited capacities. For example, a popular platform TelosB [26] has 10 kbytes data memory, 48 kbytes program memory and 1024 kbytes external flash memory (EEPROM). This requires the complexity of the algorithms in dissemination to be well fitted to the capacity profile of a sensor node. Besides, the sensor nodes generally have low bandwidth. For example, TelosB uses the Chipcon CC2420 radio and can achieve 250 kbps data rate at most. Moreover, lossy links are common in typical wireless sensor networks. This requires the routing algorithm used for forwarding data to utilize the good links other than bad links in a network; (ii) Broadcast storm. One straightforward and obvious solution for bulk data dissemination is flooding: the sink node (or base station node) broadcasts the data object to its neighboring nodes. On receiving the data, each node stores and forwards the data object with broadcasting to its neighbors. This process continues recursively until all network nodes are fully delivered. However, this approach may result in serious contention, collision and redundancy in large scale and dense networks. It is called the “broadcast storm” problem [27]. It is obvious that “broadcast storm” can greatly degrades the dissemination performance in terms of both transmission and time efficiency [28].

#### 2.2.4. Energy Efficiency

Generally, sensor nodes are battery powered. The energy consumption of each operation that relates to dissemination is listed in Table 1 [23].

Although the EEPROM write operations is very energy consuming, it is a necessity and with no space for optimization as each node should finally store the entire data object. On the other hand, radio related operations are more frequent during the dissemination and has much opportunity for optimization, especially in large scale wireless sensor networks [23,29].

### 2.3. System Overview and Taxonomy

The available literature on bulk data dissemination tackles the above challenges from different aspects. The works can be categorized according to the different optimizing goals: Works that mainly focus on ensuring eventual consistency (reliability) are listed in the first category. These works mainly include NACK-based and piggybacked ACK-based approaches. Works that mainly focus on scalability are listed in the second category. Works that mainly focus on improving the transmission efficiency of the dissemination are listed in the third category. There are tremendous works in this category and we further classify these works into three subcategories according to the employed techniques as follows: Exploiting wireless characteristics (link quality, link correlation, etc.), network coding and multi-channel. Works that mainly focus on improving energy efficiency are listed in the fourth category. These works mainly include active sleep mechanism and coordinated sleep scheduling.

**Figure 1:**
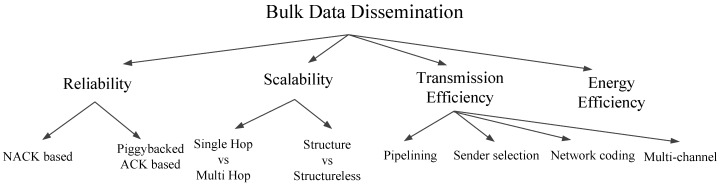
Taxonomy of data dissemination protocols.

Figure 1 shows the taxonomy in high level. For each category, we will introduce the literature of the techniques.

### 2.4. Reliability

Due to the application of bulk data dissemination such as reprogramming, 100% reliability is strictly required. Proposals mainly for ensuring the reliability of bulk data dissemination in sensor networks are listed in this category.

According to the message type (NACK or ACK) employed for reliable transmission, we further categorize these approaches into two subbranches as shown in Figure 1. ACK based mechainsms: The classic direct ACK mechanism works well in communication with unicast models: A receiver node directly replies an ACK message to the transmitter upon receiving a data message. If the transmitter node does not receive the ACK message for that packet, it retransmits the packet. This approach, when employed in wireless sensor networks with broadcast operations and very limited bandwidth, may be prone to high collision [30,31], contention [32] and possible ACK storm problem [27]. The advantage of ACK based approach is that the retransmission procedure is done by the transmitter, which does not require the receivers to be aware of the data object size in advance [33]. On the other hand, NACK based mechanisms is receiver initiated: A node reports NACKs that indicate the missing packets to its transmitter, and then the transmitter retransmits the requested data packets upon receiving NACKs. The disadvantage of NACK-based approach is that it requires the receiver to be aware of the data object in advance, which needs extra negotiation messages to inform the receivers about the data object. The advantage of NACK-based approach also comes from the awareness of the data object: it can batch up the requests for different packets in a bit-vector and send the bit-vector to its transmitter. This can greatly reduces the number of control message (ACK/NACK messages) transmissions.

#### 2.4.1. NACK Based Mechanisms

Here we survey the work attempting to ensure the 100% reliability based on NACK messages. Kulik et al. [28] proposed the SPIN (sensor protocol for information dissemination) protocol which incorporates two key innovations in their work: First, it employs three stage handshake to establish reliable dissemination.

- ADV—new data advertisement. When a node has new data to distribute, it can advertise this fact by broadcasting an ADV message containing the meta-data.
- REQ—request for data. A SPIN node sends a REQ message when it wishes to receive some actual data.
- DATA—data message. DATA messages contain actual sensor data with a meta-data header, indicating the source node id, sequence number, etc.

**Figure 2:**
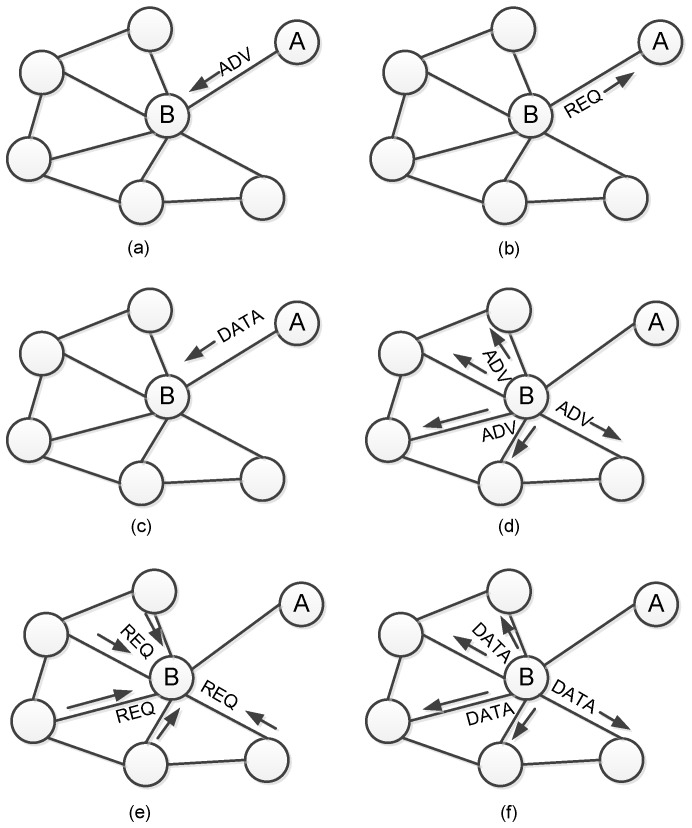
The SPIN Protocol [28]. Node A starts by advertising its data to node B (**a**); Node B responds by sending a request to node A (**b**); After receiving the requested data (**c**); node B then sends out advertisements to its neighbors (**d**); who in turn send requests back to B (**e**,**f**).

This mechanism establishes the framework for more recent reliable dissemination protocols. Figure 2 shows workflow of the SPIN protocol. Second, SPIN employs an adaptive resource management mechanism to save the energy consumption on low-power-level nodes. The mechanism simply polls the energy level of a SPIN node, and cut back certain activities when energy is low, e.g., by being more prudent in forwarding third-party data packets. The energy management, however, is not the main concern of SPIN. It just specifies an interface that application can use to probe their available resources.

In [28], a node broadcasts ADV messages upon new data arrivals. However, the frequency of ADV message broadcast is not carefully considered, which may lead to high ADV transmission overhead. With this observation, Levis et al. [34] proposed a self-regulating algorithm Trickle targeting at achieving high reliability with low control message overhead. Trickle’s basic idea is simple: For each node, ADV messages are sent with adaptive period. A node immediately broadcasts ADV messages about its received data packets when it has new data or hears ADV messages claiming old data; when a node receives a ADV messages that claims new data, it transmits REQ messages for the new data; when a node does not receive any requests for a while or receive only the ADV messages containing the same data, it increases the length of the ADV broadcast period exponentially. Besides, Trickle also incorporates a simple suppression mechanism that a node will suppress its ADV message broadcasts when hearing identical ADV messages. With this algorithm, Trickle is able to scale to thousand-fold variations in network density, quickly propagate updates, distribute transmission load evenly, be robust to transient disconnections, handle network repopulations, and impose a maintenance overhead on the order of a few packets per hour per mote.

Hui et al. followed the framework of three-way handshake used in [28,34], and makes some further optimizations in their proposal Deluge. First, Different with [28,34] where the data objects’ reliability is guaranteed all at once, Deluge divides a data object into a set of fix-sized pages with each page containing certain number of packets, and achieve the strict reliability page-by-page. This is an important optimization as for bulk data dissemination, as a Trickle or SPIN node may need to keep a very large bit sequence to record the reception for all packets of a large data object (For the case with a 10 KB data object and 23 bytes payload, a node needs an 56 bytes bit vector). The three-way handshake is slightly modified: in an ADV message, a node claims its received number of pages. When a node receives an ADV that contains more pages, it sends a REQ to that node for its next page to receive. Deluge requires the sequential order of page transmissions, such that a node only needs to contain the largest page number in the ADV messages to claim all its received pages. Second, Deluge modifies the suppression mechanism in two ways: (i) When a node hears ADV messages that contain smaller page number, it suppresses its ADV broadcast; (ii) When a node hears a data packet of its needed page, it suppresses any requests for a full round. This also suppresses other possible interfering senders. Deluge is the first protocol that considers both efficiencies of bulk data propagation and network data maintenance. It is the default bulk data dissemination and reprogramming protocol of TinyOS [35]. Many later works are built upon the framework of Deluge.

#### 2.4.2. ACK Based Mechanism

Here we describe the works attempting to ensure the 100% reliability based on ACK messages.

Inspired by the ARQ protocols in the wired network, Kulkarni and Arumugam [33] design two kinds of implicit ACK based recovery mechanism for dissemination with TDMA: Go-Back-N based recovery algorithm and Selective Retransmission based recovery algorithm. With the Go-Back-N algorithm, a sensor transmits a packet with sequence number $n_{f}$ only if it has received all packets with sequence number smaller or equal to $n_{f}$. Thus when a node transmits a packet with sequence number $n_{f}$, it provides implicit acknowledgement for all packets from 0 to $n_{f}$. To exploiting the implicit acknowledgements in the recovery, a node maintains a window of packets with sequence number $n_{ia}-1,...,n_{ia}+N$, where $n_{i}a$ is the highest sequence number for which the node has received an implicit acknowledgement from all its successors and *N* is the window size. Now, a node will forward packet with $n_{f}$ only when all its neighbors have forwarded packets with $n_{f}-\frac{N}{2}$ or higher. Otherwise, the node will start retransmitting packets within its current window. With Selective Retransmission algorithm, different with Go-Back-N, a node can transmit a packet with $n_{f}$ when not all packets with smaller sequence numbers are received. More formally, a node *j* can transmit packet with $n_{f}$ only if it has received all packets with sequence number 0,...,$n_{f}-b-1$. Hence when a node forwards a packet with $n_{f}$, it provides the acknowledgements for packets with $0,...,n_{f}-b-1$. With this observation, the authors designed a similar recovery mechanism as follows: Each node records the highest sequence number $n_{unack}$ for the unacknowledged packet. A node *j* will forward packet with $n_{f}$ only if it $n_{unack}>\left(n_{f}-b\right)$. Otherwise, node *j* will retransmit the packet with sequence number $n_{unack}$.

Liang et al. [36] targeted at establishing prompt retransmission mechanism with CSMA MAC protocol. This work (called Typhoon) follows the framework of Deluge and has two salient features: First, in order to avoid ACK storm problem, Typhoon transmits data packets via unicast. This approach allows receivers to acknowledge the receipt of individual packets and thereby enables quick recovery of lost packets. Second, to compensate for the low transmission efficiency of unicast, Typhoon also allows nodes to receive the unicast packets by snooping on the wireless medium. This is feasible as for example, the CC2420 radio provides the ability to disable *address filtering* enabling a node to receive all packets irrespective of the destination addresses. Third, the REQ messages are broadcasted periodically instead of after receiving ADV messages. This is important as the snooping cannot guarantee 100% reliability. With periodic REQ mechanism, the snooping nodes can be eventually covered by its neighboring senders.

Combining the above two techniques, Typhoon achieves prompt retransmission as well as data dilivery to all the nodes in a broadcast domain with single transmission.

#### 2.4.3. Short Summary

Table 2 shows the proposals with main focus on reliability. We can see that (1) the use of ACK/NACK is closely related to the MAC layer protocol. For networks with TDMA, where time synchronization is required, ACK is preferred since it can be used at the same for synchronization. For networks with CSMA, NACK is preferred. The reason is that NACK merges multiple packet reception/loss acknowledgements together, which can avoid the ACK storm problem and greatly reduce the transmission overhead; (2) The overhead of ADV essentially depends on the periodicity. More frequent ADVs can reduce the propagation delay, but incurring more transmission overhead; On the other hand, less frequent ADVs reduce the transmission overhead but increase the propagation delay. Trickle’s [34] ADV policy is most popular because it achieves a good tradeoff between the transmission and delay overhead.

### 2.5. Scalability

According to the network supported, these works can be divided into single hop and multi hop categories. According to the propagation manner, these works can be divided into structured (data propagates along an underlying structure) and structureless categories.

#### 2.5.1. Single-Hop vs. Multi-Hop

XNP [40] is the default reprogramming and dissemination protocol in TinyOS 1.0. It simply broadcasts a large data object to multiple receivers. This single hop design clearly cannot meet the dissemination requirement for multi-hop wireless sensor networks.

MOAP [21] supports multi-hop dissemination. The sink (sender) node initiates the dissemination by broadcasting PUBLISH messages. After receiving PUBLISH messages, a receiver unicasts a SUBSCRIBE message to the sink node. Then the sink node starts to transmit the data object. If the receivers lost some packets after a transmission round, it replies a NACK which carries the lost packets in the object. Then the sender retransmits the lost packets. When a receiver receives the entire data object, it is allowed to further forward the data object, with the same process as the above. Deluge [39] follows many design principles of MOAP, and further introduces data object segmentation to enable multi-hop pipelining and support multiple-object dissemination.

#### 2.5.2. Structured vs. Structureless

Deluge is a structureless dissemination approach. Works that follow Deluge’s frameworks are also decentralized approaches.

Differently, Naik et al. [41] proposed to construct stationary CDS (connected dominating set) structure for data dissemination. The key idea of Sprinkler is to establish the optimal structure that requires the least number of transmissions to cover all nodes, by considering the distance and network topology. The CDS nodes are one-hop away from non-CDS nodes, and are responsible for forwarding the data objects to them. It first divide the whole network into several clusters with rectangle areas, and the cluster head of each cluster is used to form a connected dominating set (CDS). We note that nodes in the CDS are connected while nodes out of the CDS are connected to at least one CDS node, and only nodes in the CDS are responsible to transmit data packets. The authors propose a location-based CDS construction mechanism, which is at most $18\frac{2}{3}$ times that of an minimum CDS. After the CDS structure, a two-phase dissemination commences. In the first phase, the sink node initiates the dissemination for all nodes in the CDS. The non-CDS nodes can also overhead data packets in this phase, but they do not actively request for the missing packets. In the second phase, nodes in the CDS recover the missing packets of non-CDS nodes with three-way handshake. During the above two phases, TDMA transmissions are employed. Besides, Sprinkler design an effective and efficient coloring algorithm to construct the CDS structure in a distributed manner. The resulting performance ratio of our algorithm is $1\frac{8}{9}$ and the time complexity is O(∆log${}^{2}$ n). Following Sprinkler, there are also other works that disseminate large data object in centralized fashion [8,42,43,44]. However, since they concentrate more on the efficiency optimization, we will introduce these works in Section 2.6.

The pros and cons of structured approaches such as Spinkler are clear. Pros: By establishing a stationary structure, Sprinkler is able to employ TDMA transmission with stringent time scheduling, reducing possible contentions and thus improving transmission efficiency. Cons: Since sensor networks are often with dynamic links, the established structure may not be efficient all the time, thus the performance may greatly degrades, especially under dense and highly dynamic networks.

### 2.6. Transmission Efficiency

Many proposals aim to improve the transmission efficiency of bulk data dissemination. There are two main aspects for optimizing the transmission and time efficiency: Single hop transmission and multi-hop transmission. For single hop optimization, the primary goal is to overcome the problem of lossy links. Typical approaches mainly include aggressively sender selection (which avoids senders with poor links), employing network coding and multi-channel transfer. For multi-hop optimization, the main goal is to increase the transmission concurrency at different hops.

As shown in Figure 1, works aiming to improve the transmission efficiency can be further categorized into four sub-categories according to the main employed techniques: Pipelining, sender selection, network coding and multi-channel. These four techniques are orthogonal, i.e., more than one techniques can be employed in a protocol. However, different works focus on refining different techniques. Some works [41,45,46,47] mainly focus on setting optimal parameters for efficient pipelining; Some works [41,42,48,49,50,51,52,53,54] are based on the pipelining, and further focus on the sender selection to utilize better nodes in the network for fast data propagation; Some works [15,54,55,56,57,58] further study the use of network coding to further reduce the retransmission overhead; Some other works [36,47,59] propose multi-channel designs to fully utilize the spectrum and enable concurrent transmissions.

#### 2.6.1. Multi-Hop Pipelining

**Figure 3:**
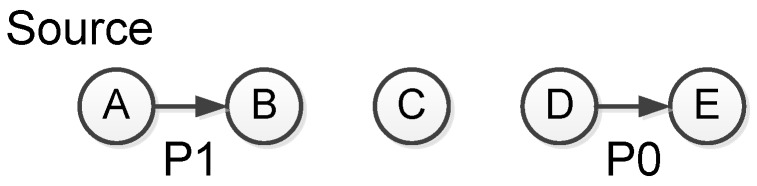
Illustration for multi-hop pipelining. A three-hop spacing is required to avoid wireless collisions [39]. As illustrated, while node A is transmitting page 1, node D can transmit page 0 at the same time.

In Deluge [39], the authors divide a data object into several pages to reduce the bit vector buffer for packet recovery as well as enable multi-hop pipelining, as shown in Figure 3. While Deluge establishes multi-hop pipelining in dissemination, it does not carefully consider the efficiency of pipelining.

Dong et al. [60] identified the tradeoff between multi-hop concurrency and negotiation overhead in the page size configuration, and then optimize the page size to achieve better performance of pipelining. The tradeoff is as follows: When the page size is small, the pages can be propagated to remote nodes more quickly, enabling more concurrent transmitters at different hops. This is expected to reduce the transmission delay; On the other hand, however, as we need to employ three-way handshake mechanism for ensuring reliability, each page transmission incurs at least two message transmission and stochastic delay. This is expected to increase the transmission delay. To optimize the page size configuration, the authors first model the relationship between page size and the dissemination delay, considering link quality and topology information. Then they found that there exists an optimal page size with a given network. As a result, by optimizing the page size, the authors can achieve the optimal performance of Deluge. The limitation is that it can guarantee good performance only under static network conditions. The pre-configured page size cannot adapt to highly dynamic network conditions.

Park et al. [45] increased the pipeline efficiency at a different angle. Generally, the approach (called GARUDA) is similar with Sprinkler [41]. They employ an underlying CDS structure and disseminate the data object in two phases. The key contribution is that they employ out-of-sequence packet forwarding during the two dissemination phases: a node that has lost a packet can continue to forward any higher sequence number packets that it might receives. Previous protocols transmit packets sequentially, which may result in that downstream network bandwidths left under-utilized when the forwarding of higher sequence number packets is suppressed in the event of a loss. As there are much fewer packets in further hops, the concurrent transmissions is inhibited and the pipelining efficiency is reduced. Differently, with out-of-sequence design, transmissions to further hops are prioritized and the pipelining efficiency is increased. A problem of this approach is the possible NACK implosion: downstream nodes will issue a chain of NACK requests for holes detected in the sequence of received packets, even when the concerned packets are not available. To deal with that, the authors require each sender to broadcast an ADV message claiming which packets it can offer and also require each receiver node to transmit NACK only when it has received the ADV messages indicating that the sender has received sufficient packets. Table 3 summarizes the proposals with main focus on the improving transmission efficiency via pipelining.

#### 2.6.2. Sender Selection

Bulk data dissemination is data intensive and based on the broadcast operation. Due to the nature of wireless broadcast, there will be collisions if the transmissions are not carefully scheduled. Further, as a sensor network is often deployed in hostile environments, the links qualities of links at different positions can be highly different. Hence, alleviating the wireless collisions while selecting the good links are important for efficient bulk data dissemination.

Sender selection is an important means of avoiding wireless collisions and the poor links in the network during bulk data dissemination. It essentially selects the nodes with more receivers and good throughput to be data forwarders, which can reduce the delay and transmissions during data dissemination. There are two main issues in sender selection: first, the metric for accurate sender estimation. This is important as the metric decides whether the most effective nodes could be evaluated with the largest impact. Second, the contention mechanism. Different with simple suppression used in Deluge, multiple potential senders need to coordinate to let the sender with the largest impact transmit first. The coordination, however, incurs extra delay. Hence it is important to select the most effective sender to transmit first with a contention mechanism.

There is a rich literature in sender selection for bulk data dissemination, and these works exploit various network information for accurate sender impact estimation and employ subtle mechanisms for efficient contention. According to the information used in the sender selection metrics, we divide existing dissemination protocols with main focus on sender selection into three sub-categories: (i) Topology(one-hop neighbor tables); (ii) Link quality; (ii) Link correlation.

Please note that the above three sub-categories follows a progressive way: the second category also exploits topology while the third category also exploits the former two information. We will also introduce the contention mechanisms in the middle of these works.

**Figure 4:**
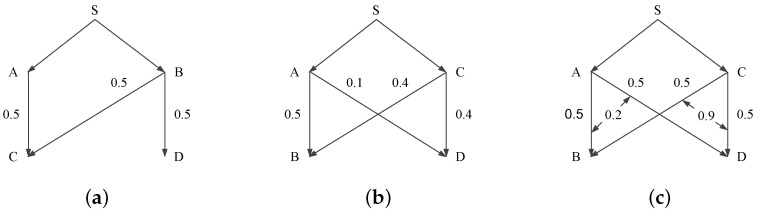
Factors that impacts the accuracy of sender selections. (**a**) Number of uncovered nodes; (**b**) Link qualities; (**c**) Link correlations.

Before going into the details, we first take a glance at the three impacting factors of sender selection in a simple topology shown in Figure 4. S is the sink node and tries to disseminate a data object to all the other four nodes. Node A and node C are responsible to forward the data object to node B and node D. The sender selection procedure is to select either node A or node C to forward the data packets. Figure 4a shows the impact of topology information (i.e., number of nodes requiring the data object). Obviously, the node with more receivers is a better sender. Figure 4b shows the impact of link quality. As wireless links are commonly unreliable [61,62], the variance in link quality can significantly impacts the data transmission efficiency. We can see that if node A transmits, only 0.5 effective receptions can be expected. Otherwise, if node B transmits, there will be 0.4 × 5 effective receptions. In brief, senders with strong outbound link qualities are more effective. Figure 4c shows the impact of link correlation. Link correlation denotes the probability that two links have the same/different packet losses (or receptions) [13,63,64]. Intuitively, when link correlation is strong, the receivers tend to have the same packet receptions and losses, which incurs less retransmissions. Otherwise, the receivers tend to have different packet receptions and losses, incurring more data retransmissions. Next, we will elaborate the related works in the three sub-categories with main focus on (1) the sender selection metrics and (2) the contention mechanism.

##### Exploiting Topology Information

Deluge essentially employs a random sender selection mechanism. The node that receives a request will try to transmit data packets immediately. When another sender hears the data packets, it will suppress its own data transmissions and wait for the next request.

Kulkarni et al. [48] first employed online sender selection for bulk data dissemination (called MNP). It exploits the one-hop neighboring information, and tends to select the sender with the most number of receivers to transmit first. The framework of the MNP protocol follows the three-way handshake mechanism of Deluge, and incorporates the sender selection into the three-way handshake as follows: Each node periodically broadcasts ADV messages, which contain the number of distinct REQ messages received since the last data transmission. Nodes that receive ADV messages carrying more pages then broadcast REQ messages. The REQ messages have two purposes: first, for requesting the data objects; second, they carry the largest REQ numbers contained in the received ADV messages. If a node hears a ADV or a REQ that contains a larger number of distinct REQ messages, it fails in the sender selection and turns off its radio from broadcasting ADV/data messages. If a node broadcasts three ADV messages in a row without receiving larger REQ numbers, then it wins the sender selection and starts the data transmissions.

The rational of MNP is clear: nodes that receives the most number of distinct REQ messages have more potential receivers, and its transmissions are therefore the most efficient. However, there are several inefficient aspects in MNP: First, different with the wired networks, wireless communications are commonly unreliable [65]. As a result, a node with the most receivers does not necessarily mean that its transmissions can be received by most receivers. E.g., if the link qualities to the receivers are bad, then the nodes that receives less REQ messages but have much better outbound links may yield better packet delivery performance. Second, though the contention mechanism is lightweight, it lacks accuracy and time efficiency. A node waits for three rounds of ADV messages before it can start data transmission, it means that a node has at most three chances to compete with other potential senders. It is possible that a sender that fails in the first round of ADV may receive the most REQ messages in the future two rounds. However, as it has turned off its radio, it cannot be estimated the most effective sender. Also, the contention needs three rounds of ADV messages, which may greatly increase the dissemination delay as the sender selection happens frequently for each transmission round and each neighborhood.

##### Exploiting Link Quality

Pradip De et al. [49] identified that link quality plays an important role in the sender selection for bulk data dissemination and proposes a dissemination protocol (called ReMo) that leverages the LQI and RSSI link estimation for more accurate sender selection. Each node in ReMo keeps two kinds of information for its neighboring nodes: (1) Link quality. As each node periodically broadcasts ADV messages, a node can obtain the LQI and RSSI information from the received ADV messages and estimate the link qualities from the ADVs’ source nodes. The link quality from node *j* to node *i*, $l_{ji}$ is estimated in Equation (1).

(1) $$lji\left(t+1\right)=\gamma lji\left(t\right)+\left(1-\gamma \right)\frac{l_{j}^{avg}\left(t\right)}{l_{max}}$$

where $l_{j}^{avg}\left(t\right)$ is the average of the LQI values of the packets received from node *j* in current transmission *t*. The weight factor *γ* decides the contribution of the previous estiamtion of the link quality.

Page download potential (PDP). Unlike Deluge, ReMo does not require the sequential order of page transmission. Hence the nodes that contains more un-received pages is more potential to be an efficient sender. The PDP recorded by node *i* for node *j*, $p_{ji}$ is denoted by:

(2) $$p_{ij}=\frac{|S_{j}-S_{i}|}{|S_{obj}|}$$

where $|S_{j}-S_{i}|$ is the set difference between the page sets of node *i* and node *j*, $|S_{obj}|$ is the total number of pages contained in the data object obj. We can see that the more different pages a node keeps, the larger of its PDP value. Next, with the above two information, node *i* can calculate a hybrid metric for its potential sender node *j* $m_{ji}$ as in Equation (3).

(3) $$m_{ji}=p_{ji}*l_{ji}$$

where $p_{ji}$ is the PDP and $l_{ji}$ is the link quality from node *j* to node *i*. After that, node *i* unicasts a REQ message the node with the largest *m* value. It is worth noting that ReMo employs two types of REQ messages: full REQ and half REQ. A full REQ from node *i* to node *j* means that the link quality from node *j* to node *i* is very good, and *j* should start data transmission when it receives the REQ; A half REQ from node *i* to node *j* means that the link quality from node *j*, though the best link that node *i* can select at the time, is not good enough. It is possible as the best sender of *i* is selected according to both link quality and PDP. Node *j* that receives the half REQ will estimate the link quality by obtaining LQI and RSSI from the half REQ message, and then decide whether to transmit the requested data packets according to the estimated link quality.

In summary, ReMo achieves more accurate sender selection metrics by considering link quality and avoid the contention by migrating the sender selection to the receiver side (The receivers decide which senders to request, nodes with poor links are less likely to receive REQ messages). The drawback is that it considers only single link and overlooked the impact of broadcast: intuitively, it is possible that in a neighborhood a node that has only one good outbound link wins the sender selection, instead of the node that has more good outbound links. It is obvious that the latter is a better sender as the data packets are broadcasted to all receivers.

Dong et al. [50] proposed a more considerate sender selection for bulk data dissemination call ECD. ECD also follows the framework of Deluge, and mainly focus on improving the sender selection in terms of both sender selection metric and the contention mechanism. Different with ReMo, ECD’s sender selection is sender initiated. Before data transmission, each sender evaluates its own impact considering all its outbound links to potential receivers (nodes that have broadcasted REQ messages in for its data object). The sender selection metric is calculated as follows:

(4) $$m{\left(ecd\right)}_{i}=\sum_{j\in N_{i}}q_{ij}$$

where $N_{i}$ is the set of node *i*’s neighboring potential receivers, and $q_{ij}$ is the link quality from node *i* to node *j*. The ECD’s metric is essentially the expected receptions at multiple receivers. Compared with the metric in ReMo [49] where a receiver only selects the best inbound links for itself, ECD considers the efficiency of the data transmission to all potential receiver nodes. In order for a rapid and scalable sender contention, ECD employ a density aware transmission prioritization mechanism. When each sender has estimated its own impact, it starts a back-off based on the metric value:

(5) $$t_{backoff}\left(u\right)=\left(N-m_{ecd}\left(u\right)\right)\delta +X$$

where *N* is the neighbor size, *δ* is a constant time unit and *X* is a random value used for contention resolution when two senders have the same metric value. We can see that the larger the impact, the shorter of the back-off timer. By such design, the sender with largest impact is most likely to transmit data packets first, i.e., wins the contention.

Jayashree et al. [54] proposed a sender selection scheme for data dissemination in mesh networks. The key idea of [54] is to select the senders likely to lead to all nodes receiving the flooded data in the least time. Each node estimates its neighboring nodes’ potential sender metrics to evaluate which node should be the sender. The metric takes into account link quality, bit-rate, number of uncovered nodes, etc. as folows:

(6) $$U_{A}\left(B\right)=\sum_{C\in N_{B}}P_{B,C,b}\cdot b\cdot I_{B,C}$$

where $U_{A}\left(B\right)$ is the estimated value of node *B* at node *A*, $P_{B,C,b}$ is the link quality from node *B* to its neighboring node *C* under bit rate *b*, *b* is the current bit rate and $I_{B,C}$ is a flag indicating whether node *B* has useful packets for node *C*.

When applied in sensor networks, where bit rate is constant (250 Kbps), the metric is similar in principle with ECD’s metric. The reason is that (i) for each different potential sender, *b* is the same and (ii) a ECD node *i* accounts for only the link qualities to nodes requesting data packets, which means *i* definitely have useful packets to those nodes. Similarly, UFlood explicitly marks a flag to decide whether to account for a node. As a result, both ECD and UFlood only account for the link qualities to nodes that request data packets. The difference with ECD is that UFlood is designed for mesh networks and can frequently exchange feedbacks. Further, it uses multi-generation network coding to reduce data retransmissions.

##### Exploiting Link Correlation

While the above approaches have demonstrated the effectiveness of link quality in achieving communication efficiency, further improvement has been hampered by the assumption of link independence. Recently, Srinivasan et al. [13] discovered that the packet receptions on different links with the same source are not always independent. Further, they propose a novel metric *κ*, which is the normalized Pearson’s coefficient, to characterize wireless link correlation and show that link correlation can have a large impact on the performance of broadcast based protocols such as flooding and opportunistic routing (e.g., Deluge and ExOR [66]). For example, when link correlation is largely strong in the network, Deluge’s performance is much better than that with weak link correlation.

Based on the above observation, Zhu et al. [51] designed a novel sender selection scheme for efficient flooding (Though it is not a work originally for dissemination, the design of the sender selection can be as well directly employed in dissemination). Similar with ECD [50], the sender selection metric is also an indication of the number of uncovered nodes. Note that an uncovered node is a node that has not received the data object. The difference is that the metric calculation combines both link quality and link correlation as shown in Equations (7)–(9).

(7) $$m_{cf}\left(u\right)=\sum_{k\in U(u)}L\left(u,k\right)\cdot \left(1-CP_{u}\left(k\right)\right)$$

U(u) is the set of uncovered nodes, L(u,k) is the link quality from node *u* to node *k* and $CP_{u}\left(k\right)$ is the coverage probability from node *u* to node *k*, which is further calculated with link correlation.

$CP_{u}\left(k\right)$ is calculated accumulatively as Equation (8) and link correlation is indicated by the conditional packet reception probability (CPRP) as shown in Equation (9).

(8) $$CP_{u}\left(k\right)\leftarrow 1-\left(1-CP_{u}\left(k\right)\right)\cdot \left(1-P_{v}\left(k|u\right)\right)$$

The term $\left(1-CP_{u}\left(k\right)\right)$ is the probability that *k* had not received the packet *M* before *v*’s forwarding; the term $\left(1-P_{v}\left(k|u\right)\right)$ is the probability of *k*’s failure to receive *M* from *v* given the condition that *u* received *M*. As a result, Equation (9) is the probability of node *k* is being covered either by (i) previous transmission in the network or (ii) current forwarding from *v*.

The measurement and calculation of link correlation need a little more explanation: Each node periodically broadcasts beacon messages (with sequence numbers) and use bit sequences to record the packet reception/lost, where a “1” denotes a received message and a “0” denotes a missed message. Each node also exchanges the bit sequences with its neighboring nodes. Then node *v* can calculate the link correlation between link v→u and v→k as follows:

(9) $$P_{v}\left(k|u\right)=\frac{\sum_{i=1}^{w}B_{vk}\left(i\right)\&B_{vu}\left(i\right)}{\sum_{i=1}^{w}B_{vu}\left(i\right)}$$

where $B_{vk}\left(i\right)$ is a bit representing node *k*’s reception status of the *i*th beacon messages sent from node *v*. The result is the probability that *v* receives a packet given that node *u* receives the same packet. With the above formulations, a node can obtain the sender’s impact, i.e., the expected number of uncovered nodes.

Next, a contention mechanism is required to ensure the node with the largest impact starts the data transmission first. The proposed solution is similar with that of ECDs: a back-off timer is employed, where the durations are set according to the impact value as follows:

(10) $$T_{backoff}\left(u\right)=\frac{C}{TE(u)}$$

where *C* is a constant value. Compared with ECD, the back-off timer design cannot effectively differentiate senders as when the senders’ impacts are very large, their back-off timers will be very close.

Guo et al. [52] considered a different scenario where low duty cycle is enabled. Low duty cycle is often employed for energy conservation, as a node turns off its radio when there is no traffic and turns on the radio when it has packets to transmit/receive. The authors propose a novel flooding protocol called Correlated Flooding, which exploits the link correlation information to establish a fixed structure and the duty cycles for all sensor nodes, and then floods data packets along with the structure and the duty cycles.

Correlated Flooding first estimates the link quality and link correlation of neighboring nodes, and then group the nodes with strong correlation together and assign the corresponding slots (nodes within the same group wake up at the same time slots). For each group, only the node with the worst link quality replies ACKs, and its ACKs can represent other nodes receptions within the same group. Similar with [51], before the grouping, each node periodically broadcasts beacons with sequence numbers, and records the receptions and losses with bit sequences. Differently, the calculation of link correlation incurs much more overhead. When a node collects all bit sequences (representing the beacon message receptions/losses) of its next-hop nodes, it groups the bit sequences with k-means method, using the hamming distance as the distance parameter in k-means. Once each node has grouped its next-hop nodes, it broadcasts a message claiming the grouping results of its next-hop nodes, i.e., informing the next hop nodes about which groups they should belong to. When a node receives multiple grouping messages, it should select which group it belongs to. The principle for group selection is that a node tries not to be the worst link node in that group. If it is the worst link node in all potential groups, it selects the sender with the best link quality. After the group selection, a node broadcasts its selection for keeping a consistent view at the sender side. When the grouping is done, nodes within the same group have the same wake-up duty cycles, and only the worst link node replies ACKs during the flooding.

Although this work establishes a structure before data transmission, the idea is essentially to select nodes with strong outbound link as senders. As the data packets transmissions follows the structure and corresponding schedules, the sender contention mechanism is not required.

Zhao et al. [8] observed when a structure like [52] is established, the performance of bulk data dissemination can be further improved in the following two important aspects: (1) improving the data/page transmission mechanism and (2) reducing the considerable negotiation overhead. The authors proposed a novel protocol called CoCo to optimize all procedures in structured dissemination.

CoCo first concluded that there are three phases in bulk data dissemination with correlated structures: (1) Structure establishment; (2) Data streaming; and (3) Data recovery. For structure establishment phase, CoCo follows the key idea of [52] and further incorporates the coordinated sleep scheduling [42] to optimize energy consumption. Time is divided into three slots: sleep, transmitting and receiving. When sender nodes are in transmitting state, its child nodes are in receiving state and its upstream (parent) nodes are in sleep state. By coordinated scheduling, core-nodes have 1/3 sleep time while leaf (non-core) nodes have 2/3 sleep time. This greatly helps reducing energy consumption. For data streaming phase, different with previous works, CoCo does not require entire page-by-page transfer, but requires page-sized transfer. The key insight is that when transmitting a page, upstream nodes often have more stored data pages. While retransmitting, all previous works only transmit the missing packets of requesters, which greatly derogates the bandwidth utilization. Differently, in each transmission round, a CoCo node can merge packets from different pages and transmit one-page-sized packets to fully utilize the link bandwidth. For the data recovery phase, since each node has fixed parent and child nodes in the structure, ADV packets are eliminated. Each node only replies REQ packets at the end of each transmission round. As the page-by-page transmission is changed, the format of the REQ packets is also different. It contains a starting packet position and a page-length bitmap indicating the packet receptions for a page sized packets following the starting packet position, e.g., a REQ like “3|10011” means this bitmap indicates packet 3–8 with packets 5 and 6 lost. Compared to the previous solutions, CoCo gives a holistic design, which optimized all three phases of bulk data dissemination. CoCo+ [44] extended the design of CoCo and adopted energy saving techniques tailored for structured dissemination.

Table 4 summarizes the proposals with main focus on improving transmission efficiency via sender selection.

#### 2.6.3. Network Coding

The basic principle of network coding is to send different encoded combinations of packets to the receivers (called encoded packets). The receiver can recover all the packets by solving linear equations when it receives enough different combinations. Rateless codes provide an efficient means of addressing channel contention in sensor networks, while at the same time reducing control messages [67]. The nature of rateless codes is interesting: receivers do not need to indicate which specific packets require retransmission; instead, they just need to receive sufficient number of encoded packets, which can be used to decode the original packets. Thus, rateless codes can be used to reduce communication and energy consumptions.

Hagedom et al. [55] proposed the first work Rateless Deluge to exploit rateless codes for enhancing the dissemination performance. They modifies the original Deluge [39] protocol in that it supports random linear coding (a kind of rateless codes) for data transmission. The modification mainly lies in the transfer mechanism with respect to the request mechanism and the data transfer. (1) The change to the request mechanism is straightforward. When using rateless codes, a receiver can decode the whole page when sufficient number of encoded packets are received. As a result, only the number of missing encoded packets must be transferred in the REQ message, which greatly reduces the REQ message size compared to original Deluge (which needs to contain a long bit vector indicating the missing packets within a page); (2) The change to the data transfer is substantial. In Deluge’s transfer mechanism, a sender transmits data packets according to the bit vectors in received REQ messages. When there is a missing packet in its received REQ message, it retrieves a packet from the flash memory and transmit it. This is infeasible for rateless implementation because the entire page is required before the encoding process. Similarly, a receiver node cannot decode the original data page before it receives sufficient number of packets. Rateless Deluge then requires the entire page reception before decoding/encoding. It also provide a pre-coding mechanism to optimize the decoding delay: when the page is not entirely received, a node can precode the received packets and waits for the remaining packets to finish the encoding process. Compared with Deluge, Rateless Deluge performs much better in networks with poor link qualities (packet reception ratio below 0.5).

The drawback of dissemination based on rateless codes is that it incurs considerable encoding and decoding delay. Extensive efforts have been done to reduce the encoding/decoding overhead [15,56,57,58].

Hou et al. [15] identified that the coding performance is closely related to the network dynamics and propose an adaptive coding algorithm for bulk data dissemination. The coding methodology is randomly generate *N* coefficients and compute the linear combination of *N* packets. For different link densities, the optimal value of *N* (which produces the least packets) is different. Generally, if nodes have more neighbors, they can encode more packets together without losing reliability since they can get enough combination from their neighbors to decode. Based on this observation, the authors propose an adaptively determining coding scheme as follows: Each node records a avgNeighbor to indicate its long-term number of neighbors. avgNeighbor is calculated accumulatively as avgNeighbor=α·avgNeighbor+(1−α)·curNeighbor, where curNeighbor is the number of neighbors in current time. When a node is about to start transmission, it decides *N* according to the average neighbor size avgNeighbor. By storing the matchup of avgNeighbor and *N* in the sensor node, the computational overhead is greatly reduced.

Rossi et al. [56,68] further enhanced the encoding/decoding performance used for bulk data dissemination. While Rateless Deluge [55] and AdapCode [15] are based on rateless codes on Galois fields of size $2^{q}$ with $q=8(GF\left(2^{8}\right))$ and $GF(2^{5})$, in Synapse and Synapse++ [56,68], packets are encoded using fountain codes with GF(2), i.e., by means of bitwise XOR operations, which greatly reduces the encoding and decoding overhead.

Although the use of fountain codes greatly reduces the coding overhead, the performance is still far from satisfactory. All the above coding-based dissemination protocols still have two deficiencies: (i) They all require Gaussian elimination for decoding, which is expensive in terms of computation time; (ii) They cannot adapt to different network densities. Especially, in dense networks with high link variance, the performance will degrade greatly [57]. The reason is that when a node has to cover both good links and poor links, most of the delay and transmission overhead is caused by the poor links. It is better to let other nodes to cover those with poor direct links. Based on these observations, Dong et al. [57] employed simple yet efficient XOR coding algorithm, which further reduces the decoding overhead; and also dynamically adjusts the inter-page waiting time according to the network density (Recall that bulk data they are based on the framework of Deluge) to achieve good performance under different network conditions. The rationale of the latter improvement is that by decreasing the interpage waiting time for fast propagation in dense and diverse link conditions, nearby nodes (with good link qualities with the source) can be used as forwarders to further away nodes (with poor link qualities with the source), which reduces the overhead on poor links.

Gao et al. [58] used a relatively simple idea to further reduce even avoid the decoding overhead. The key idea is simple: exploiting the concurrency ability (multi-thread) of sensor nodes to perform decoding operations while transmitting/receiving packets. Different with the above proposals, MT-Deluge uses an incremental decoding algorithm to enable packet-level decoding (previous approaches support only page level decoding) and greatly enhances the propagation. Besides, MT-Deluge also incorporates an adaptive page size which can dynamically changes according to different link qualities. When link qualities are good, the page size is set long while when link qualities are poor, the page size is set short.

Table 5 summarizes the proposals with main focus on improving transmission efficiency via network coding.

#### 2.6.4. Exploring the Synergy among Link Correlation and Rateless Codes

Since the exploitations of link quality, link correlation and rateless codes are separately, extensively explored, the design tradeoffs among these three impacting factors are yet to be addressed. As reported in [13], rateless codes perform better when links are negatively correlated. We can infer that there exists an overlap between link correlation and rateless codes when used for optimization of bulk data dissemination.

Iftekharul et al. [53] followed the idea of the above work and propose a novel dissemination protocol that considers both link correlation and network coding. Syren simply combines link correlation and rateless codes, but ignores the design tradeoffs between link correlation and rateless codes. We believe there exists opportunities for further improvements considering the tradeoffs among these three factors.

#### 2.6.5. Channel Diversities

There are also tremendous works using channel diversity techniques to enhance the dissemination performance, such as multi-channel and constructive interference.

##### Multi-Channel

**Figure 5:**
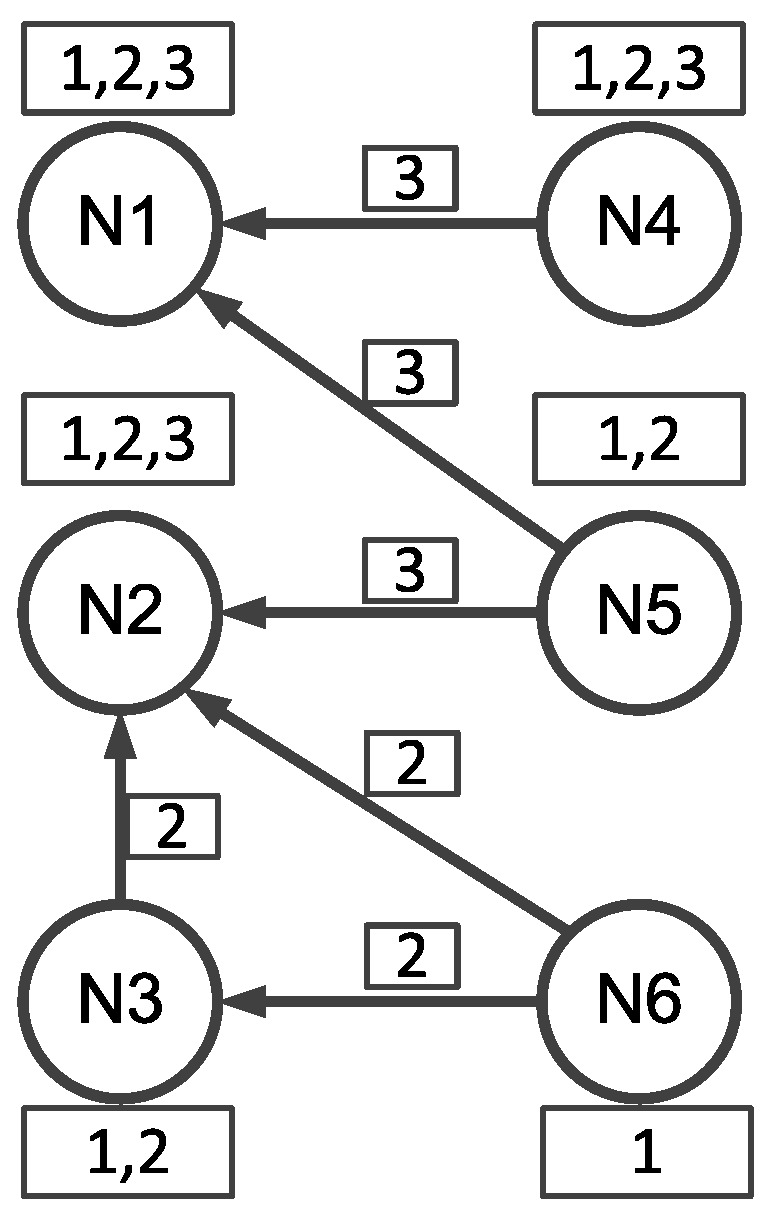
An example of the Gappa protocol [59]. A circle denotes a sensor node while a rectangular over(or below) a circle denotes the received pages of that node. An arrowed edge from N1 to N2 denotes that N2 receives a REQ message from N1. A rectangular besides an arrowed edge indicates the requested page number.

Wang et al. [59] exploited multi-channel technique to enable transmission concurrency in a neighborhood of a network. Basically, Wang et al. [59] are based on the framework of Deluge, where a data object is segmented into pages and three-way handshake is employed for ensuring reliability. The key idea of [59] is to assign each data page a fixed channel, thus enabling the concurrent transmissions of different pages in a neighborhood. The working logic of this work is shown in Figure 5.

Initially, all the sensor nodes are communicating in the same channel (called control channel) and try to select one sender for each different page transmission (in corresponding transmission channel). The sender selection mechanism is very similar with MNP [48] (As discussed in Section 2.6.2) and is different with it in an important way. In MNP, only the node with the largest number of REQ messages can start transmitting data pages; while in [59], as each page is to be transmitted in different channels, there is at least one sender for each data page and corresponding channel. In a neighborhood, some nodes are requesting (broadcasting REQ messages) for page *i* and some other nodes are requesting for page *j* (j≠i). Potential senders that intend to provide transmissions for the same page (*i* or *j*) compete to be a sender for that page. Similar with MNP [48], each node contains the number of distinct received REQ messages ReqCtr in the REQ and ADV messages for contention. A potential node fails the sender selection when it overhears a ADV/REQ message intending for the same page and with a ReqCtr larger than its own ReqCtr. Otherwise, if a node broadcasts ADV messages three times, it wins the contention and starts data transmissions. Before changing the communication channel, a selected sender first broadcasts SwitchChannel messages to inform the receivers that has requested for its data page. When a node receives the SwitchChannel and identifies that its requesting page is about to be broadcasted in that channel, it changes its channel to the same channel with the sender. The channel for page *i* is decided by chn=i%16 where chn is the channel for the coming transmissions, 16 is the number of non-overlapping channels that the CC2420 radio can provide and % is the modulo operation [69], such that different pages can be transmitted in different channels at the same time. After the page transmission, a sender broadcasts EndDownload message to inform receivers to return into the default control channel. Note that the control channel can also be used for data transmission as it also corresponds with certain page numbers.

By such design, for each different requested page in a neighborhood, there is one sender transmitting the data packets in that page in the prescriptive channel. For example shown in Figure 5, N4 and N5 are requesting page 3 and N6 is requesting page 2. N1 receives two REQs for page 3, N2 receives one REQ for page 3 and two REQ for page 2 and N3 receives one REQ for page 2. For page 3, N1 has the most number of distinct REQs and wins the contention for transmitting page 3. Then N1,N4 and N5 will change communication channels to be channel 3 as 3%16 = 3. (For CC2420, the channel will be 13 as the channel sequence is from 11 to 26) Similarly for page 2, N2 wins the contention and changes to channel 2 for data transmission. N3 and N6 also changes to channel 2 for receiving data packets of page 2. After the sender selection, we can see that N1 and N2 are transmitting different pages at the same time, which can obviously improve the dissemination efficiency.

**Figure 6:**
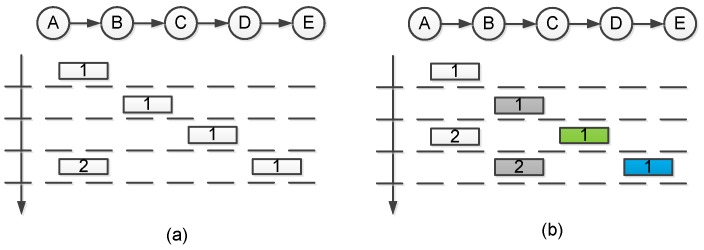
(**a**) Propagation of consecutive pages on a linear topology when only one frequency channel is used. Notice that node A has to wait until time period 4 to transmit the second page in order to avoid colliding at B with node CÂąÂŕs transmission of the first page; (**b**) When nodes can use different frequency channels to transmit data packets (indicated by different colors in the figure) the wait time is reduced by one time period.

Different with [59], Liang et al. [36] exploited multi-channel for bulk data dissemination in another way: they employ multi-channel transmissions to avoid intra-flow interference and improve the efficiency of multi-hop pipelining. As shown in Figure 6a, in traditional pipelining with single channel, concurrent transmissions at different hops can occur at least two hops away. Otherwise, there will be intra-flow collisions (If node A start transmitting page 2 at time slot 3, the concurrent transmissions of node A and node C will collide at node B due to the hidden terminal problem [70]). With multi-channel pipelining (Figure 6b), only one hop spacing is required to avoid transmission collisions. To fully exploit the concurrent transmissions at neighboring hops, the best way is to synchronize the transmissions at different hops such as TDMA scheduling [71,72]. However, as bulk data dissemination generally requires very high reliability, the ACK overhead is unacceptable. As a compromising approach, the authors employs different timer designs to prioritize the transmissions at further hops rather than the nearer hops to the source node. For nodes that just finishes transmitting a page, the timer for broadcasting ADV message is prolonged (a random value between [15, 25] ms). For other nodes, the timer for broadcasting ADV messages is much shorter (a random value between [0, 10] ms). For example in Figure 6b, when A finishes transmitting page 1 to B, its ADV timer becomes longer than B’s. Hence B will advertise earlier than A and is more likely to start transmitting page 1 to C (instead of node A transmitting page 2 to node B).

##### Constructive Interference

Ferrari et al. [18] showed that constructive interference is practical in sensor networks. They observed that there is a high probability that the concurrent transmissions of the same packet will result in constructive interference if the temporal displacement of these transmissions is less than 0.5 μs. This observation enables the later works exploiting constructive interference in various protocols [14,47,73,74].

**Figure 7:**
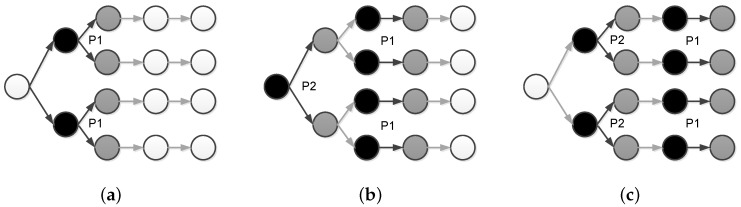
Illustration of pipelining over a dissemination tree [47]. (**a**), (**b**), and (**c**) denote the three consecutive transmission rounds. The black nodes denote the senders, the grey nodes denote the receivers and the white nodes denote the idle nodes.

Doddavenkatappa et al. [47] identified and exploited the opportunity to exploit constructive interference for efficient bulk data dissemination. The work (called Splash) first establishes dissemination tree to enable tree pipelining, which exploits constructive interference to effectively create parallel pipelines over multiple paths. The tree pipelining is shown as in Figure 7. The black nodes are transmitting nodes while the grey nodes are receiving nodes. The white nodes are nodes that have not yet received any packets. The node in the left is the sink node and prepares to transmit a data object to all other nodes. A data object contains multiple packets such as P1, P2, etc. Initially, the sink node starts transmitting packet P1 to level 1 nodes. Level 2 nodes then concurrently forwards P1 to level 3 nodes to form constructive interference (Figure 7a). Similarly, level 3 nodes starts forwarding P1 to level 4 nodes. At the same time, the sink node starts to transmit P2 to level 1 nodes. Note that at different level, the transmitting channel is different in order to enhance the pipelining performance (Figure 7b). This process continues to the further levels of the dissemination tree (Figure 7c).

However, merely constructive interference cannot guarantee the dissemination releasibility. To achieve this end, the authors uses several other optimizing techniques as follows. First, they allow opportunistic overhearing. When a node misses a packet, it still have opportunity to receive the same packet from other nodes in the same level. Second, as reported in [74], the number of concurrent senders is a deciding factor to the performance of constructive interference. Deriving the optimal number of concurrent senders is time consuming, which may in turn affects the temporal displacement of concurrent transmissions. Instead, the authors exploit the transmission density diversity for more reliable concurrent transmissions, i.e., transmitting the full data object twice using different number of concurrent senders. This approach is shown to be effective in their experiments. Third, since the two times of transmissions have delivered some packets to all nodes, Splash performs a third-time full data object transmission using XOR coding. In their experiment conducted in Indriya [75] and Twist [76], 90% nodes are fully covered by the first three rounds. Fourth, for the left nodes, Splash uses local recovery mechanism to recover the missing packets. The local recovery mechanism uses three-way handshake to 100% ensure the reliability.

Overall, Splash incorporates most of the optimizing techniques mentioned in above sections and yields the best performance in the state of the art. The limitation of Splash, however, is perhaps the exploitation of density diversity can be closely related to the environment and network topology, which makes the first two transmission rounds prone to packet losses. Also, it is possible that in some network conditions, the first round can achieve almost perfect reliability, leaving the second and third transmission rounds a waste of energy.

Summary of the approaches with main focus on transmission efficiency. All existing approaches can be categorized into two branches. The first one is to exploit wireless dynamics such as link quality, link correlation, and constructive interference, etc. The second one is to design subtle mechanisms to fully exploit the network capacity, such as consecutive transmission, network coding, multi-channel transfer, etc. When new wireless phenomena are observed, novel mechanisms are proposed to exploit them for improving dissemination efficiency. Future directions of protocol designs depend on the exploration of wireless dynamics and new requirements of bulk data dissemination.

#### 2.6.6. Sender Selection vs. Constructive Interference

We notice that sender selection and constructive interference are two main means for collision avoidance. Sender selection allows only one sender in a interfering neighborhood, while constructive interference requires all senders transmit the same packet at the same time. The benefit of sender selection is that it is lightweight and scalable. It is lightweight because only one sender is required to transmit. It is scalable because it does not have any requirements on network topology or transmission time/sequence. On the other hand, the benefit of constructive interference is that it strengthens the wireless signals, enhancing the wireless communication range and quality. For scenarios where nodes are placed near and are with strict time synchronization, constructive interference significantly improves the protocol performance. However, the strict time and topology requirements makes it available for only specific scenarios and hard to co-exist with other protocols like CTP [77].

### 2.7. Energy Efficiency

Energy consumption is a critical issue for resource constrained sensor nodes as bulk data dissemination is often used for software update, bug fix, surveillance video distribution, etc. [78]. Although the above works improves transmission efficiency, which at the same time can save energy consumption by reducing the number of transmissions, Mainwaring et al. found that radio idle listening is the major source of energy waste [79]. There is also a rich literature with main focus to achieve energy efficiency by reducing the idle listening time. In this section, we will introduce some representative designs and compare the pros and cons of them.

**Figure 8:**
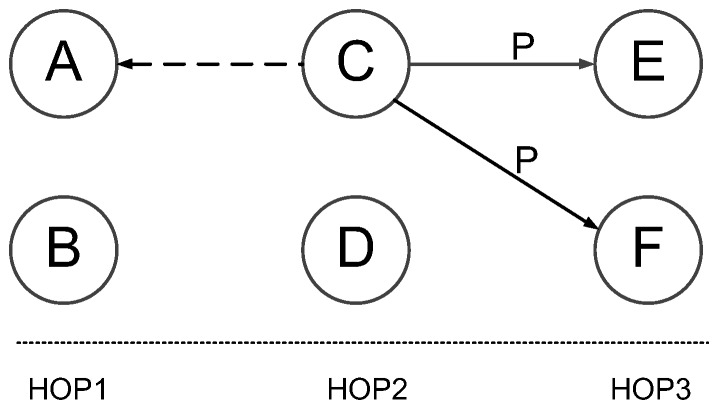
Illustration of energy saving mechanism in [33].

Kalkarni et al. [33] designed a probabilistic approach to reduce the active radio time with TDMA scheduling (called Infuse). As discussed in Section 2.4.2, Infuse use the forwarding of the successors as implicit ACKs, this in turn requires the predecessors to keep radios active when it is in their successors’ transmission slot. This is clearly not a wise design in the view of energy efficiency. To deal with this problem, the authors propose a probabilistic model for energy saving: each successor node selects a preferred predecessor node and include its node ID in its forwarding packet. When a predecessor node identifies that it is preferred by some successor node(s), it keeps the radio active in the successors’ transmission slot as it is responsible for recovering possible packet losses at these nodes. Otherwise, the predecessor node turns off its radio with high probability to save energy consumption. As Figure 8 shows, node A and node B are both predecessor nodes of node C and node D and only node A is preferred node. When node C forwards data packet P to its successor nodes E and F, node A should keep its radio on to overhear the packet P as an implicit ACK. At the same time, node B turns its radio off in this slot to save energy.

This approach is the state-of-the-art work for optimizing energy consumption in bulk data dissemination with TDMA. It (1) avoids ACK storm and (2) reduces the energy consumption for overhearing implicit ACKs. However, it can be used only with TDMA, which is not common, especially in nowadays large scale sensor networks.

In the work MNP [48], energy is also a main concern. The energy conservation scheme is combined with the sender selection mechanism: when a node loses the sender selection contention, it sets a sleep timer and turns into sleep state (turns off its radio). More specifically, the sleep timer is proportional to the size of the new program, and lasts for approximately the expected code transmission time. Although such design can reduce the active radio time and save energy consumption, it has side effects. First, it could incur the unfair sender selection. As a node will turns off its radio immediately when it identifies another node with more REQs, it cannot continue to collect the follow-up REQ messages. This may result in inaccurate sender impact estimation. Second, the sleep timer is a crucial issue. If the timer is longer than necessary, the dissemination delay will be prolonged.

**Figure 9:**
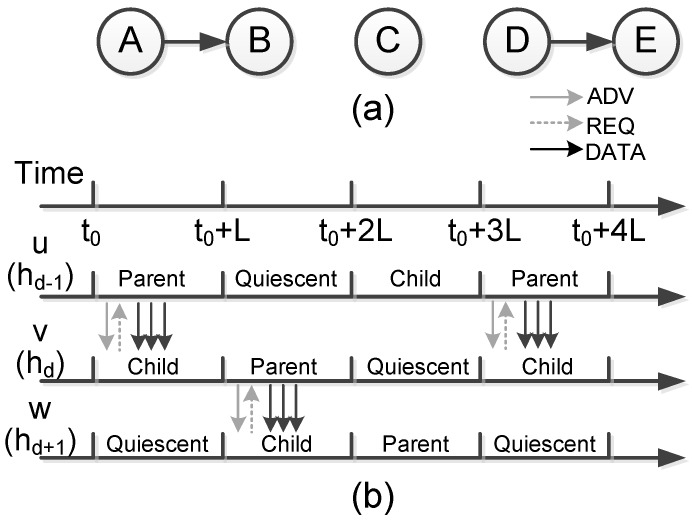
Illustration of energy saving mechanism in [42]. (**a**) Pipeline with single channel; (**b**) The coordinated scheduling.

Huang et al. employed a coordinated scheduling scheme in [42] for energy efficient bulk data dissemination. The work (called cord) establishes an underlying connected dominating set (CDS) structure before a two-phase dissemination commences. During the dissemination, like TDMA, time is divided into three recursive slots: P, C and Q slots. In a P slot, a node performs as a parent node and transmits data packets to its downstream nodes. In a C slot, a node performs as a child node and receives data packets from upstream nodes. Note that for a CDS node, it can reply REQ messages to request missing packets during the C slot, while for a non-CDS node, it cannot transmit until the CDS nodes have received the whole data object. In a Q slot, a node turns off the radio. The three slots can be seamlessly integrated with the multi-hop pipelining. As multi-hop pipeline requires a three hops spacing in order to avoid intra-flow collisions (as discussed in Section 2.6.1), the nodes between the concurrent transmissions (e.g., node C and node F in Figure 9a) should not transmit any packets. CORD’s key idea is to turn off the radios of these nodes to save energy without affecting the data propagation. As shown in Figure 9b, For non-leaf nodes, the slot schedules are P-Q-C. For leaf nodes, the slot schedules are P-Q-C. When a node is in P slot, its child nodes are in C slot such that they can communicate. The transmission mechanism follows the design of Deluge (page segmentation and ADV-REQ-DATA three way handshake). The slot length is set according to the page size, and is approximately the transmission time of a page (including the control message exchanges). Theoretically, for a non-leaf node, the energy reduction should be close to $\frac{1}{3}$ while for a leaf node, the energy reduction should be close to $\frac{2}{3}$.

All the above works have the assumption that the network is with always-on radios. However in general, a typical sensor network often employs low duty cycle (or low power listening, LPL) MAC layer protocols [80,81,82] in order to save energy consumption [9,83,84]. Although the above mentioned approaches can effectively reduce the energy consumption during the dissemination process itself, the problem of combining energy efficient dissemination with a practical low-power running sensor network is yet to be solved. For example, Deluge will send more than 100 times packets over a low power link layer than over an always-on link layer (as reported in [85]).

Gao et al. [85] proposed a dissemination protocol built on low power link layer. Overall, the protocol is a combination of always-on dissemination and LPL MACs: when the dissemination is in negotiation process (exchanging control messages such as ADV or REQ), the network is in LPL mode. While when a data page is about to be transmitted, the senders and receivers enter always-on mode to consecutively transmit a batch of data packets. There are two important practical issues in this work: First, the senders should be aware of the receivers wake-up schedules to transmit data packets when the receivers are awake. Second, when applied in LPL, a sender will transmit tremendous controls messages before its receivers wake up, resulting much unnecessary control message overhead and long dissemination delay. To overcome the above two problems, the authors first employ a self-suppression scheme to prevent too many senders at the same time. The key insight of the self-suppression scheme is to adjust the probability of control message transmission according to the estimated number of potential concurrent senders. The more potential senders, the less control messages are needed as the probability that receivers can overhead ADV messages are high. Then an adaptive sleeping scheme is employed to reduce unnecessary energy consumption and keep the consistency of low power settings. The key insight is that when there are few or no ADV or REQ messages received, the length of duty cycles can be augmented to save more energy. The adjustment of the cycle length is carefully designed in order to avoid lost links (a sender can never transmit a packet to a receiver).

#### Short Summary

All existing works try to reduce the radio-on time (reducing transmissions and increasing the radio-off time) for energy optimization. The reason is that for current sensor platforms such as Mica and Telos motes, wireless communication is the main source of energy consumption. However, there are two main concerns for the energy consumption issue of bulk data dissemination: (1) Co-existence with other common protocols such as CTP [77]. Many designs change the duty cycles to save energy consumption, however, there may be underlying protocols such as low-power-listening (LPL) [9,80] which manages the duty cycles. This conflict should be addressed before the energy optimizing (2) Support for real time applications. Some WSNs are designed for real time applications such as fire alarm. As a result, the duty cycling may cause extra delay to the data transmission and alarm information dissemination.

## 3. Open Issues

In this section, we first summarize the above works in high-level and discuss the key insight of these works. Then we discuss the future directions of dissemination protocols in two ways. First, the potential optimizations motivated by the recent advances in wireless communications such as cross-technology interference/communications [86,87], error estimating codes [17,88] and constructive interference [89]. Second, the design paradox from the system view such as the co-existence with the existing protocols, the transparency over MAC layer protocols and the specific applications. These open issues are likely to be utilized to achieve more efficient and generally applicable dissemination protocols in various network scenarios.

### 3.1. Summary of the Literature

The basic principal for the existing works on dissemination optimization is three-fold:

1. Towards exploiting the spatial wireless diversity. For example, the sender selection schemes try to exploit the best sender for each round of transmissions in the dissemination process. Besides, constructive interference is also based on the appropriate relative positions between the concurrent senders, which also tries to exploit the spatial opportunities for dissemination.
2. Towards exploiting the temporal wireless diversity. For example the protocols [50] detects the time varying link quality and choose the time with the best link quality for transmission.
3. Towards exploiting the wireless channel diversity. The works that uses multichannel techniques try to find the best strategy for wireless channel reuse to achieve high-efficiency bulk data dissemination.

We can see that by exploiting various techniques, the reliability/scalability/efficiency are largely improved. However, as we will discuss in the following sections, there are still much space left to be optimized.

### 3.2. Modeling of Dissemination Performance

Though many works are working on optimize the dissemination performance, few works have been done to discuss the optimality of dissemination. Dong et al. [60] modeled the performance of bulk data dissemination and discussed the optimality that can be achieved. However, the modeling assumes that the data propagates along the shortest path in the network. As we have introduced in Section 2, many works do not follow the shortest path. For example, the work [10] uses constructive interference and the resulting propagation path is most likely to be different with the shortest path. Li et al. [90] present a model that considers link characteristics in the dissemination process. However, they also assume the shortest path propagation. Therefore, a model that can adapt to different sender selection schemes, transmission schemes and negotiation schemes is left to be developed. With the model, we can theoretically evaluate the existing works and identify to what level dissemination protocols have been optimized and how much space is left to be further optimized.

### 3.3. Cross-Technology Interference and Communications

More and more wireless devices are pouring into our daily life [91,92]. These devices use different technology for communication such as Wi-Fi, BlueTooth and ZigBee. The communications can cause significant interference to low power wireless sensor networks especially in the indoor environment [92], which is termed as “cross-technology interference (CTI)”. The dissemination efficiency in sensor networks can be easily affected by CTI.

Some CTI aware transmission mechanisms have been proposed to improve the transmission performance under CTI. Using the in-packet patterns, REPE [93] utilizes the in-packet errors for adaptive retransmissions. CARE [94] uses the in-packet RSSI samples to infer the byte errors caused by CTI and apply forward error correction for error recovery.

However, these works are designed for single link transmission and cannot be directly applied for bulk data dissemination under CTI. As a result, the performance of dissemination under CTI can be further improved if the following challenges are addressed.

1. Appropriate negotiation mechanism for CTI aware dissemination is required. The existing works apply FEC codes for error recovery, which do not support reliable broadcast since each link has a different error rate and the coding is likely to be inefficient.
2. Hardware support. The existing works on CTI require high-granularity measurement of in-packet byte-level samples, which requires a >32 KHz timer. Another alternative is to use data-driven analysis to infer the in-packet error patterns [95,96] and then apply the recovery codes tailored for dissemination.
3. Coding schemes that support batch transmissions. The existing works recover errors packet-by-packet. However in dissemination, the data packets are often transmitted in unit of pages. Therefore, if page-level recovery code can be implemented, dissemination can benefit from the advances in CTI.

### 3.4. Error Estimating Codes

Parameters in dissemination protocols such as page size and coding scheme are often determined by the link level error rate [97]. The existing dissemination protocols often uses the simple accounting method for measuring link error rate, which is time consuming and often inaccurate. Recent works [17] have proposed a novel Error Estimating Code (EEC) to measure link error rate in a lightweight and real-time manner. EEC can be used to obtain the real time link error rate, which supports adaptive adjustment of the dissemination parameters.

### 3.5. Constructive Interference

Several dissemination works [47,74] have already employed constructive interference to improve the protocol performance. The CI-based dissemination approaches can achieve much better performance than the traditional dissemination. However, the requirement of stringent time synchronization makes CI difficult to be applied in practical scenarios. A potential alternative is to either lower the requirement of time synchronization for CI or design novel mechanisms for time synchronization.

### 3.6. Co-Existence with Other Network-Layer Protocols

Few of existing works assume the existence of other network layer protocols. However, in practical WSN-based systems, the network is performing data collection all the time. The co-existence of these protocols needs to be carefully studied. One possible direction is to establish a prioritization scheme, which can suppress the regular data tasks when dissemination starts. However, such alternative may cause significant delay in the routing protocols. Another alternative is to employ the capture effect [98] to support concurrent transmission to different network areas. The challenge is to select appropriate concurrent senders according to the dissemination and routing task requirements.

### 3.7. Transparency over MAC Layer

Many works [53,68] are designed in 2.5 layer (in the middle of MAC and network layers). These works make routing decisions, as well as modifying the MAC layer parameters for optimization. However, in practical WSN-based systems, MAC layer protocol cannot be changed, otherwise, all other network layer protocols cannot work well.

### 3.8. Specific Applications

Recently, other than the general WSNs such as environment monitoring, battlefield surveillance, etc., there are more and more WSNs deployed for specific applications such as smart building [99], health care [100], underwater WSNs [101], urban air quality monitoring [102], etc. These works may have totally different occasions with the general WSN assumptions. For example, in urban air quality monitoring system, all nodes become mobile, where almost all existing works cannot work well.

We can see that, existing dissemination protocols have been extensively studied for general WSN scenarios. However, to deploy them in real-world WSN-based systems, there are still many practical issues to be solved.

## 4. Conclusions

Data dissemination protocols have been extensively studied in recent years. In this paper, we present a comprehensive review of existing dissemination protocols, summarizing useful techniques employed by these works. The novel category can (1) help unveil the key technologies proposed in dissemination protocols that can be used for various scenarios and (2) help explore the designing space for the dissemination protocols. We also discuss promising future directions in the perspective of practical deployment and the optimization chance brought by the advances in wireless communications. The novel techniques in wireless communications can be potentially employed to improve the dissemination performance in the future work.

The authors declare no conflict of interest. The funding sponsors helped revising the writing of this paper.

## Figures and Tables

**Table 1:** Energy required by some operations employed in dissemination.

Operation	nAh
Receive a Packet	8.000
Transmit a Packet	20.000
Idle-Listen for 1 ms	1.250
EEPROM Read data (per byte)	1.111
EEPROM Write/Erase data (per byte)	83.333

**Table 2:** Summay of proposals with main focus on reliability.

Protocols	Platform	DataObj Aware	MAC Layer	Negotiation Messages	Control Mess- Age Transmission	Other Aspects
Infuse [33]	MICA2 [37] TelosB [26] XSM [38]	No	TDMA	Implicit ACK	Broadcast	Out-of-order transmission
Typhoon [36]	Unknown	No	TDMA	Active ACK Periodic REQ (page level)	Unicast ACK Broadcast REQ	CDS structure
SPIN [28]	None	Yes	CSMA	Periodic ADV Active REQ (page level)	Broadcast ADV Unicast REQ	Negotiation suppression
Trickle [34]	MICA2	Yes	CSMA	Periodic ADV Active NACK	Broadcast	ADV suppression
Deluge [39]	MICA2 TelosB	Yes	CSMA	Periodic ADV Active NACK	Broadcast ADV Unicast NACK	NACK suppression

**Table 3:** Summray of proposals with main focus on improving transmission efficiency via multi-hop pipelining.

Protocols	Fineness	Recovery Mechanism	Pipeline Spacing	Transmission Sequence	Strucure
Deluge [39]	Page level	NACK	3 hops	In-sequence	None
Modeling [46]	Page level (with optimal page size)	NACK	3 hops	In-sequence	None
Sprinkler [41]	Packet level	Piggybacked ACK	3 hops	In-sequence	CDS
GARUDA [45]	Packet level	Piggybacked ACK	3 hops	Out-of-sequence	CDS
Typhoon [36]	Page level	NACK	2 hops	In sequence	None
Splash [47]	Packet level	NACK (in recovery phase)	2 hops	Out-of-sequence	Tree

**Table 4:** Summay of proposals with main focus on improving transmission efficiency via sender selection.

Protocols	Platform	Information Used	Contention Mechanism	Topology	Initiation
Deluge [39]	MICA2 TelosB	None	REQ suppression	Dynamic	Receiver
Sprinkler [41]	Mesh nodes	Location	Not addressed	Fixed	Pre-selected
MNP [48]	MICA2	one-hop topology	Message exchange	Dynamic	Sender
CORD [42]	MICA2 TelosB	one-hop topology, PRR	Pre-scheduled	Fixed	Pre-selected
Remo [49]	MICA2	RSSI, LQI	REQ suppression	Mobile dynamic	Receiver
ECD [50]	TelosB	one-hop topology, PRR	Back-off	Dynamic	Sender
Collective Flooding [51]	MICAz	one-hop topology, PRR link correlation	Back-off	Dynamic	Sender
Correlated Flooding [52]	MICAz	one-hop topology, PRR link correlation	Message exchange	Fixed	Pre-selected
SYREN [53]	TelosB	one-hop topology, PRR link correlation	Back-off	Dynamic	Sender
UFlood [54]	Mesh nodes	one-hop topology, PRR link correlation, bit rates	Message exchange	Dynamic	Sender

**Table 5:** Summay of proposals with main focus on improving transmission efficiency via network coding.

Protocols	Coding Strategy	Decoding Delay	Memory Overhead	Other Focus
Rateless Deluge	Random linear code	6.96 s	2.44 KB	Up to 20 pkts per page on TelosB motes for acceptable decoding delay
Synapse	Fountain code	462 ms	1.5 KB	Employing ARG optimization
ReXOR	XOR	184 ms	1.59 KB	
AdapCode	Linear Combination	430 ms	1.4 KB	
UFlood	Random network coding	>7 s	>5 KB	The overhead is acceptable for Mesh networks
MT-Deluge	Random linear coding	2.08 s	2.44 KB	Using Multi-thread to improvethe concurrency of decoding and receiving
Splash	XOR	184 ms	1.76 KB	
